# Monitoring temporal hazardous air pollutant gaseous generation in ethanol plants: An FTIR-based measurement methodology

**DOI:** 10.1016/j.mex.2026.104049

**Published:** 2026-07-17

**Authors:** Mahyar Mahdavi, Bruce Dvorak, Ashraf Aly Hassan

**Affiliations:** a Department of Civil and Environmental Engineering, University of Nebraska-Lincoln, Lincoln, NE, 68583, USA; b Civil and Environmental Engineering Department, United Arab Emirates University, Al-Ain P.O. Box, Abu Dhabi, 15551, United Arab Emirates

**Keywords:** Fermentation emissions monitoring, Volatile organic compounds, Hazardous air pollutants, Portable FTIR, Dilution-Based sampling method

## Abstract

Reliable, portable, and real-time measurements of Volatile Organic Compounds (VOCs) and Hazardous Air Pollutants (HAPs) from industrial ethanol fermentation remain a challenge due to the complex gas mixture in emissions, which includes high moisture content, elevated CO_2_ concentrations, and trace gases at low concentrations. Conventional Continuous Emissions Monitoring Systems (CEMS) are typically installed downstream of air treatment units and therefore cannot capture source-specific emissions or temporal dynamics within fermentation units. To address these limitations, this study presents a portable, field-deployable methodology using Fourier Transform Infrared Spectroscopy (FTIR) for upstream, high-resolution monitoring of fermentation emissions. This method integrates commercially available instrumentation with a custom-designed dilution system and field procedures. This methodology enables high-resolution, continuous measurement of ethanol fermentation off-gases under industrial operating conditions and provides a practical approach to source-specific emissions monitoring for research, diagnosis, and optimization purposes.

•Portable FTIR configured for continuous, source-specific measurement of multi-compound fermentation off-gases.

•A nitrogen-based dilution system was developed to extend FTIR measurement capability to measure high- and low-concentration compounds simultaneously.

•A field-ready sampling procedure suitable for industrial ethanol fermentation emissions monitoring and other complex emission sources.

## Graphical abstract

**Figure:**
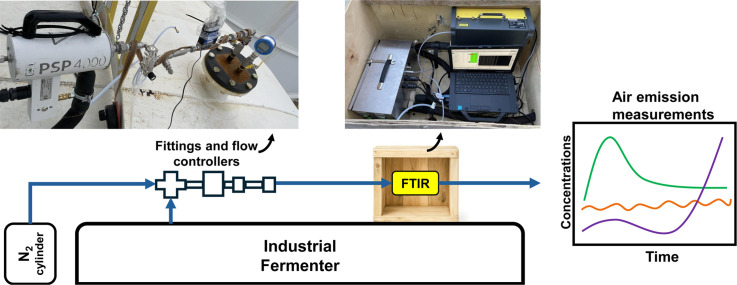

## Specifications table

**Table:** 

**Subject area**	Chemical Engineering
**More specific subject area**	Air emissions monitoring
**Name of your method**	Portable fermentation off-gas measurement
**Name and reference of original method**	U.S. Environmental Protection Agency – Method 320
**Resource availability**	none

## Background

Bioethanol is a renewable energy source considered less environmentally damaging than fossil fuels [1,2]. However, the production of bioethanol raises environmental concerns, including emissions of Volatile Organic Compounds (VOCs) and Hazardous Air Pollutants (HAPs). VOCs and HAPs are emitted at every stage of fermentation, and one particularly dominant byproduct of concern is acetaldehyde [3]. In the United States, HAPs emissions from industrial plants are regulated by the United States Environmental Protection Agency (EPA). Ethanol production facilities in the US are permitted to emit up to 25 tons per year for all HAPs and 10 tons per year for single HAPs, which is acetaldehyde, a human carcinogen that is tightly regulated by EPA, in almost all ethanol plants [4,5]. Currently, ethanol plants use CO_2_ scrubbers to treat fermentation off-gases and thermal oxidizers to treat the off-gases from the distillers’ grains dryers [6].

Emissions change over the course of the fermentation process, and hence, real-time, reliable measurements of air emissions are important for compliance and optimization purposes. Also, since air treatment processes in ethanol facilities are water- and energy-intensive [7], optimizing them can significantly reduce the environmental impacts and costs associated with ethanol production.

Air emissions from ethanol plants have high humidity, high CO_2_ content and low concentrations of VOCs and HAPs. All these features make the real-time and simultaneous measurement of these emissions challenging. There are multiple technologies for VOC measurement, but not all of them can be used to monitor air emissions from ethanol production. Table S2 compares common gas analysis technologies and their features. Based on a comparison of gas analysis technologies, the only device capable of detecting the concentrations of multiple compounds (10 or more), providing portable measurements for field use, and continuous measurement (measuring concentrations at least every minute) is Fourier Transform Infrared Spectroscopy (FTIR).

The most frequently used system for continuous emissions monitoring in ethanol facilities is a FTIR-based Continuous Emissions Monitoring System (CEMS) that measures emissions after treatment units such as scrubbers, Thermal Oxidizers (TOs), and Regenerative Thermal Oxidizers (RTOs). CEMS is frequently used for regulatory purposes. However, it is expensive for both capital and operational purposes and is usually restricted to measuring the combined off-gases post-treatment, which makes it difficult to identify the role of each unit in the overall air emissions [8].

This study employs portable FTIR to monitor emissions upstream, prior to treatment, a deviation from conventional CEMS usage that better enables understanding of the contributions of separate units and temporal changes in air emissions. This method collects real-time, source-specific data during key fermentation stages. The proposed temporary and portable emission measurement method can be valuable for diagnostic and optimization purposes, providing more detailed insight into the processes at a fraction of the cost of conventional methods. On average, the total operational cost of the portable FTIR is around 10% of the cost of CEMS, including capital costs and annual maintenance expenses. This research seeks to:

1. Establish a portable monitoring procedure, adapting existing methods for measuring high and low concentrations of off-gases from the ethanol fermentation process in the field.
2. Assess and illustrate the capability and efficacy of portable FTIR for continuous, real-time, and site-specific VOC and HAP monitoring during ethanol fermentation.

### Method details

This study adapts existing methods to a new application by using commercially available equipment. Most portable and handheld devices cannot simultaneously measure a wide range of chemical compounds with high moisture content. Approaches that can handle these types of emissions are considered semi-portable, as they are usually installed in a large, enclosed trailer or in a containerized lab. This study aims to address this gap. The development and application of a novel methodology for ethanol plant air emission monitoring required careful consideration of instrument configuration, validation protocols, and field sampling procedures. This section outlines the materials and methods used to establish and validate the measurement approach, including the FTIR setup, determination of the dilution factor, and field equipment setup. It is important to note that this method, or any alternative method not referenced by the EPA, cannot be used for official reporting. It must be validated under EPA Method 301 for each specific application and formally approved by the relevant regulatory authority as an alternative test method [9].

### Portable FTIR setup

In this study, a portable FTIR (Gasmet DX4000) was selected for its ability to measure multi-compound gas streams continuously. The selected device features a rugged design suitable for field sampling. The FTIR is paired with a gas sample probe and sampling system (M&C PSP4000) that features heated lines, heating the inlet gas to 180°C to prevent condensation inside the FTIR. The device comes with a manufacturer-supplied custom library calibrated to measure specific compounds based on the device configuration and internal component settings. Details about the library that was used for this study are shown in the supplementary materials Table S1. The FTIR uses a software (Calcmet) to analyze sample spectra and provide concentration results in real time. For this study, measurements were performed continuously in one-minute intervals to produce high-resolution results. Before each data collection run, a background measurement was performed using pure nitrogen in order to establish reference spectrum before measuring fermentation off-gases. The device was tested in the lab using gas cylinders to confirm its capability and sensitivity in measuring a mixture of nitrogen/formaldehyde. The device accurately measured 40 ppm of formaldehyde within the manufacturer's calibration curve range.

### Dilution system and tests

**Fig. 1:**
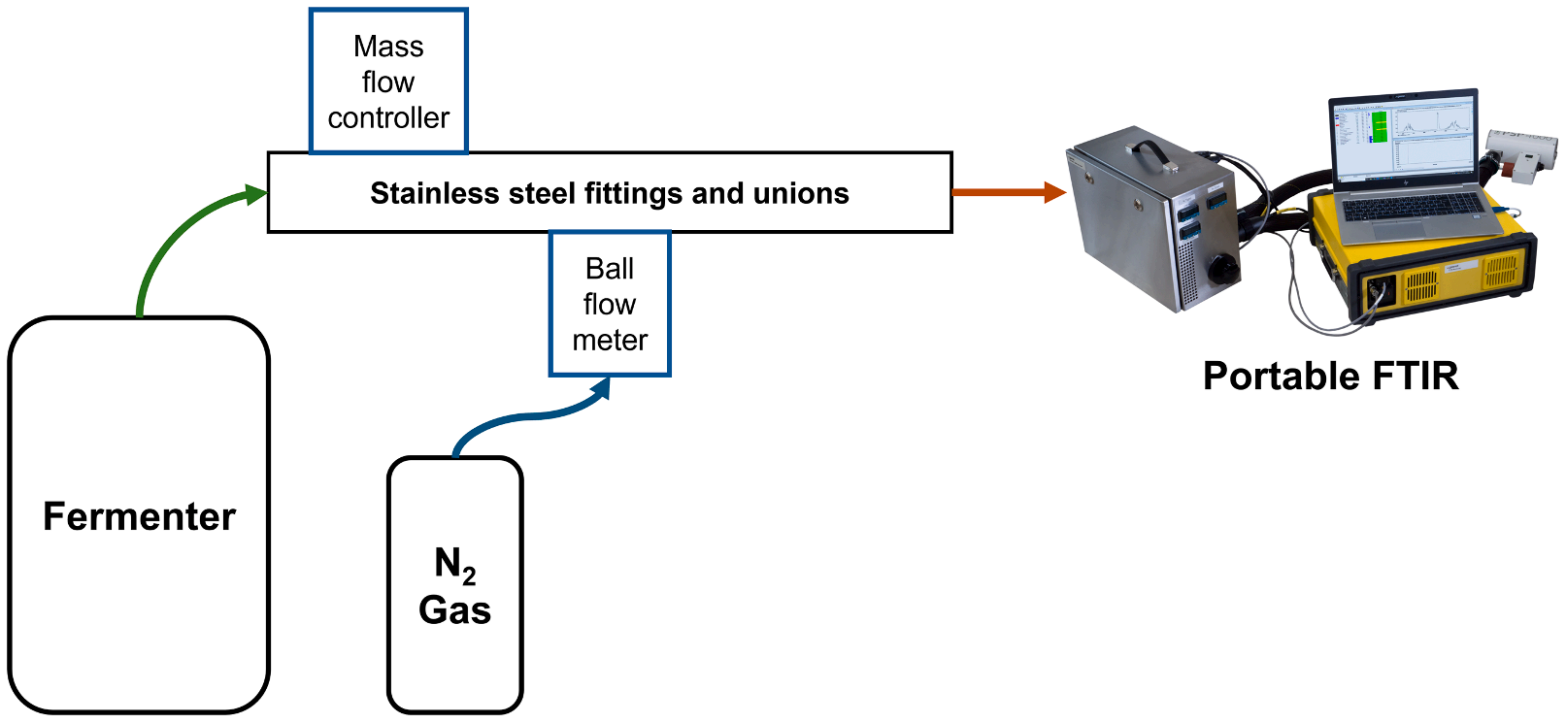
FTIR sampling setup.

The FTIR was tested on a pilot-scale fermenter in the University of Nebraska-Lincoln Chemical Engineering Process Laboratory to evaluate its performance in measuring emissions from the fermentation process. The emissions from fermentation contain high concentrations of CO_2_, water vapor, and ethanol, as well as low concentrations of HAPs and VOCs. The device was calibrated to measure low-concentration compounds (HAPs and VOCs) in the ppm range, with CO_2_ up to 20% (v/v) and ethanol up to 200 ppm, both several times lower than the actual concentrations in the fermenter gas. These high concentrations caused spectral interferences in the FTIR, which made a dilution step necessary for data collection. An initial attempt was made to obtain a new calibration library from the manufacturer; however, it was determined that the device can only be configured for either high- or low-concentration measurements, as the internal optics and mirrors are designed for a specific range [10]. Consequently, the only viable solution was to develop a dilution system using pure nitrogen gas to dilute high-concentration compounds to the device's measuring range. A line of stainless-steel tube fittings and unions was designed to mix the fermenter gas and nitrogen gas before entering the device. Fig. S1 in the supplementary materials shows a diagram of the stainless-steel tube fittings and unions used for this purpose. A mass flow controller (Omega FMA-2606A) was used to log the fermenter gas flow, and a ball flowmeter (Omega FL-2014-SS) regulated the nitrogen flow to calculate the accurate dilution factor. Fig. 1 shows the setup for the FTIR device used for data collection.

Multiple dilution factors were tested to determine the optimal flow ratio for dilution using 99.9% CO_2_ and pure nitrogen gas cylinders in the lab. During the dilution tests, the nitrogen flow was maintained at 3 L/min, as the FTIR requires a minimum of 3 L/min to operate continuously. All tests were conducted in duplicate to ensure the reproducibility of the results.

### Pilot-scale tests

The validity of the developed method and dilution system was assessed by measuring emissions from a high-volume pilot-scale fermenter at the University of Nebraska-Lincoln Industrial Agricultural Products Center, under simulated industrial fermentation conditions. Mixed corn mash containing 36% solids, yeast, and enzymes was sourced from a nearby ethanol production facility and transferred from a working fermenter to the lab. The mash was loaded into the pilot-scale fermenter with a working volume of 35L (INFORS HT Techfors-S pilot bioreactor) and continuously stirred at 400 rpm using an overhead stirrer. The fermenter temperature was maintained below 33°C throughout the experiment.

All the off-gases generated during fermentation were directed to the dilution system, and gas flow from the fermenter was logged every minute using the mass flow controller. The fermenter gas was diluted with pure nitrogen gas at 3 L/min, and the FTIR data were collected at 1-min intervals. Since the FTIR sampling system operates with a pump at 3 L/min during continuous measurement, a drop in inlet flow below this threshold, due to condensation or an obstruction within fittings, could induce a vacuum inside the fermenter. To mitigate this risk and ensure operational safety, a Tee connector was installed in the fitting line to allow ambient air to enter the system if a flow restriction occurred, preventing pressure imbalance or vacuum formation.

### Field sampling setup

In ethanol plants, fermenter tanks are connected through shared header lines, and all emissions are collected and directed to air treatment units using a piping system. Additional emission sources, such as beer wells and storage tanks, are also connected to this piping network. These connections mix emissions between tanks and cause backflow when a tank experiences a pressure drop or empties. This creates complexity for collecting data from industrial fermenters. To address this complexity for the field sampling from industrial operating fermenters, two sampling points were deliberately selected: 1) collecting data from a single fermenter tank directly and 2) collecting data from a combination of four fermenter emissions from the main header immediately upstream of the air treatment unit. During the field tests, it was observed that cold temperatures can cause condensation in the fermenter gas, restricting the flow into the FTIR device. To resolve this challenge, a heating tape (BriskHeat XtremeFLEX HSTAT) was wrapped around the fittings to prevent condensation. It was found that the heating tape alone, without additional insulation, failed to adequately heat the tubing or prevent condensation in unheated areas. To resolve insufficient and uneven heating, tubing insulation was added to envelop the heating tape and fittings, providing stable, uniform heating. Fig. S2 shows the fittings setting with and without insulation.

The sampling point for the single fermenter data collection was at the top of the fermenter, which was approximately 10 meters above ground. Environmental factors, such as wind, sun, and rain, necessitated the construction of a housing unit for the device. A wooden box was built with proper inlets and outlet holes for air circulation. The portable FTIR was fixed inside the apparatus and left at the sampling point for at least 40 h, measuring gas compounds every minute. A nitrogen gas cylinder was left at the bottom of the fermenter and connected to the fittings with Teflon tubing. The outlet gas from the FTIR had high concentrations of CO_2_ and carcinogenic compounds, such as acetaldehyde and formaldehyde, exposure to which can cause dizziness and long-term detrimental health effects. To overcome this issue, a pump with air respirator face masks (Allegro 9210-02) was used to provide clean air from the bottom of the fermenter to operators near the FTIR device. A full-body harness (3M DBI-SALA) was also used to provide fall protection for the operators at the top of the fermenter. Fig. S3 in the supplementary materials shows the sampling point in the field setup, collecting data from a single fermenter in a working ethanol plant.

## Method validation

### Dilution tests results

Table 1 shows the results from the dilution factor tests. In Table 1, the measured CO_2_ is the concentration reading from the FTIR after mixing the gases. Theoretical Dilution Factor (DF) is the dilution factor calculated based on the flow rates of nitrogen and CO_2_. The observed dilution factor is the dilution factor calculated based on the measured CO_2_, not the flows. Based on EPA Method 301, section 10.3, the ratio of expected to measured values, or the ratio of observed DF to theoretical DF, is called the Correction Factor (CF). According to EPA Method 320, Section 13.4.2, it is acceptable if 0.7 ≤ CF ≤ 1.3. For the most accurate results, the analytical results of the test method are multiplied by the correction factor [9,11]. The results from Table 1 show that the CF for this dilution methodology is within the range specified by the EPA, and this method was considered valid for further emission measurements using the FTIR.

**Table 1:** Results from the dilution tests using pure nitrogen and CO_2_.

Test number	1	2	3	4
N_2_ Flow (L/min)	3	3	3	3
CO_2_ Flow (L/min)	1.41	0.95	0.48	0.29
Measures CO_2_ (Vol%)	31.03	23.76	13.37	8.35
Theoretical DF	3.13	4.16	7.25	11.28
Measured CO_2_ multiplied by theoretical DF (Vol%)	97.03	98.77	96.93	94.19
Observed DF	3.22	4.20	7.47	11.97
Ratio of observed DF by theoretical DF (CF)	1.03	1.01	1.03	1.06

CO_2_ was selected for dilution factor tests because it is the dominant compound in the fermentation off-gas and has concentrations above the measuring range of the FTIR. Therefore, CO_2_ provided a practical basis for evaluating the nitrogen dilution system. Potential accumulation and condensation of VOCs in the fermentation off-gas, which can affect the final measured concentrations, were addressed by using the heating system to ensure all gaseous compounds remain in the gas phase and that volume-based dilution is valid.

### Field data collection results

**Fig. 2:**
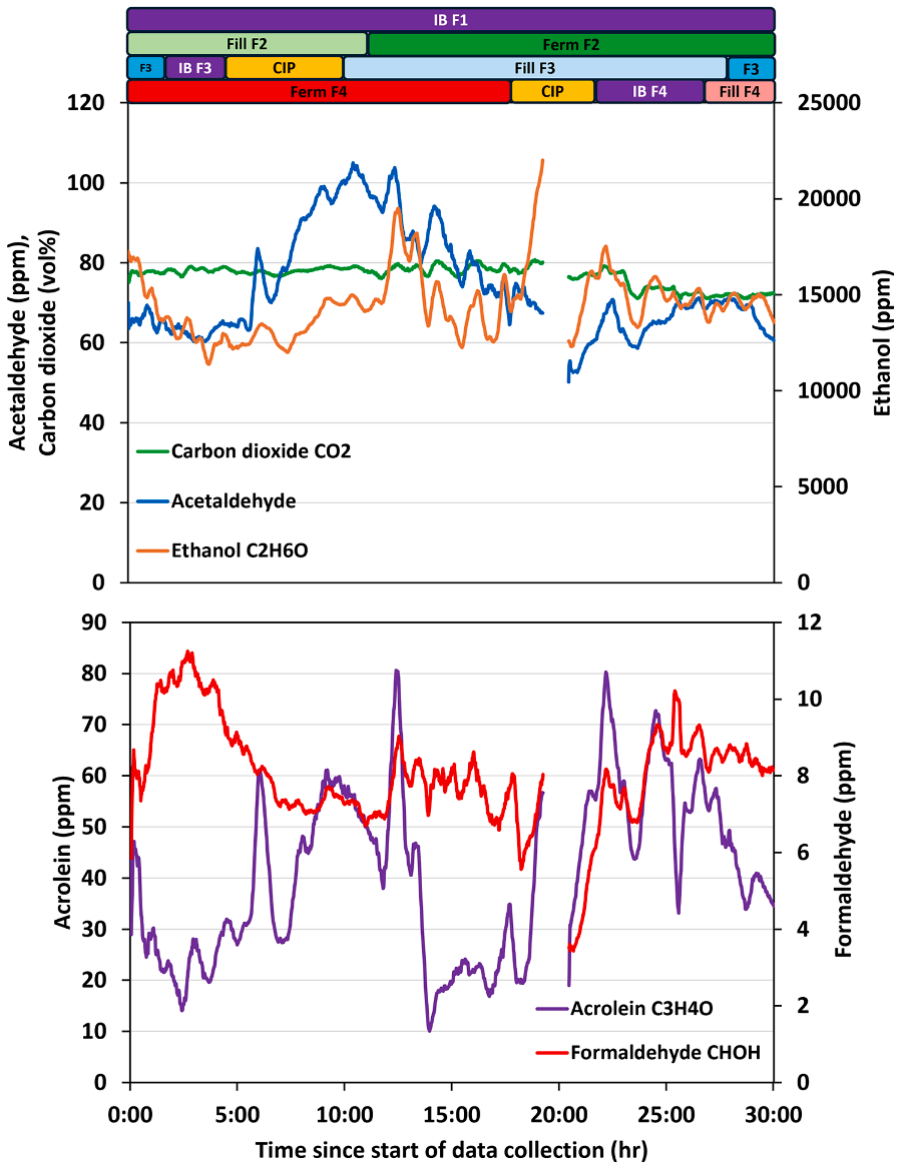
Results from fermentation emissions data collection from four operating fermenters.

The results of field sampling of the combined emissions from four operating fermenters before entering the scrubber are shown in Fig. 2. Data collection was conducted continuously for 30 h. Fig. 2 shows the gaseous compounds with the highest concentrations from the fermentation off-gases. On top of Fig. 2, the fermentation stages for each of the four fermenters are displayed. The four operational stages include filling (Fill), active fermentation (Ferm), clean-in-place (CIP), and the inactive period between batches when the fermenter is empty (IB). It was observed that the method enabled continuous data collection at 1-min intervals, and the dilution systems worked well. The results show that the method is valid for measuring fermentation emissions and was used for subsequent data collection sessions. Fig. 2 shows distinct VOC patterns that are related to the plant operation schedule and can be linked to different fermentation stages in different fermenters. The gap in the data corresponds to the period when measurements were stopped to replace the nitrogen cylinder and check the fittings and lines for possible condensations. More results are presented in a short communication paper that was submitted for publication separately. The FTIR results were compared with CEMS data from the ethanol facility, and the overall trends and removal efficiencies were consistent with the CEMS data.

## Limitations

The proposed method was developed for measurements in an industrial setting, where operational factors may affect the accuracy of the results. One of the most crucial considerations is the plant layout and the piping configuration that directs off-gases from the fermenters to the air treatment units. In an ethanol plant, there are usually 3 to 10 fermenters operating simultaneously and are connected through headers. Fermenters follow a cycle of filling, fermentation, emptying, and clean-in-place (CIP). These fermentation stages can create positive and negative pressures inside the fermenter, affecting the flow rate of the emissions and, in turn, the headspace of nearby connected fermenters. When sampling from a single fermenter or unit, sampling points should be carefully selected based on the plant layout to mitigate the effects of pressure changes from other operating fermenters. It is important to maintain a stable flow rate to the FTIR device, and adding a pump can help maintain the required flow for the measurements. It should also be noted that collecting data during CIP may damage the FTIR due to the extremely high humidity. It is best to halt the data collection during this stage.

Another consideration is the access point to the selected sampling point. In most cases, the best sampling point in a single fermenter is the headspace above the fermenter, which is a limited, sloped surface. It is important to have a container for the device that can protect it from environmental factors, and all other safety considerations should be assessed before data collection.

Another limitation is the nitrogen cylinders used for dilution. In this study, nitrogen cylinders with a volume of 8.5 m^3^ were used, each lasting approximately 30 h of continuous data collection. Another backup cylinder was used for the remaining measurements. The size of the cylinders should be selected based on the duration of data collection and transportation safety. A nitrogen generator can also be used for dilution, which is a safer option compared to cylinders but requires additional power and is less portable.

## Ethics statements

None.

## CRediT author statement

Mahyar Mahdavi: Conceptualization, Investigation, Validation, Visualization, Formal analysis, Writing - Original Draft. Bruce Dvorak: Methodology, Validation, Supervision, Writing - Review & Editing. Ashraf Aly Hassan: Methodology, Validation, Supervision, Writing - Review & Editing.

## Declaration of competing interest

The authors declare that they have no known competing financial interests or personal relationships that could have appeared to influence the work reported in this paper.

## Data Availability

No data was used for the research described in the article.

## Appendix Supplementary materials

Image, application 1
