# Supplementary material for: Monitoring temporal hazardous air pollutant gaseous generation in ethanol plants: An FTIR-based measurement methodology
Source: MethodsX. 2026 Jul 17;17:104049. doi: 10.1016/j.mex.2026.104049 (PMC13452388; doi:10.1016/j.mex.2026.104049)
Supplement: Supplementary file 1 [file mmc1.docx]

**Table S1.** Manufacturer-supplied custom library

| Component | Total Range | Lower Detection Limit (ppmv) |
| --- | --- | --- |
| H_2_O vapor | 40 vol% | - |
| Acetaldehyde | 500 ppm | 0.092 |
| CO_2_ | 20 vol% | - |
| Ethanol | 500 ppm | 0.176 |
| Formaldehyde | 223 ppm | 0.044 |
| Methane | 100 ppm | 0.037 |
| Acrolein | 200 ppm | 0.087 |
| CO | 1000 ppm | 0.079 |
| Ethane | 100 ppm | 0.054 |
| Ethylene | 100 ppm | 0.162 |
| Nitrous Oxide | 102 ppm | 0.008 |

This table lists the LDL (Lower Detection Limit) for each component, as measured individually in a balance containing only N_2_, with a DX4000 FTIR at 180° C, over 1-minute measurements. It is very important to note that, in a real sample, the presence of other IR-absorbing gases will increase the lower detection limit above the one provided above.

**Table S2.** Feature comparison of gas-stream analysis technologies

| Group  Feature | Mass-Spectrometry (PTR-MS, SIFT-MS, CIMS, IMS) | Infrared/Laser Absorption (FTIR, TDLAS, CRDS, DOAS) | Photoacoustic (PAS) | Gas Chromatography (GC) | Non-Dispersive IR (NDIR) | Photoionization Detector (PID) |
| --- | --- | --- | --- | --- | --- | --- |
| Detection Principle | Ionize, separate by mass or mobility | Measure molecular absorption bands | Measure pressure waves from modulated light | Separate by volatility/boiling point, detect by FID/ECD/MS | Measure absorbance at a single IR wavelength | Ionize compounds with UV lamp and measure current |
| Portability | Yes (IMS, presence alarm, no accurate concentration) | Yes | Yes | No | Yes | Yes (handheld CO₂/CH₄ meters) |
| Continuous Measurement | Yes | Yes | Yes | No (Batch runs, ~2–15 min per chromatogram) | Yes | Yes |
| Multi-Compound Capability | Yes (Moderate, ~5–20 species) | Yes (High, tens of species per full-spectrum scan) | No (~2–8 target gases) | Yes (High, can separate and quantify dozens per run) | No (Single gas per optical filter) | No (Aggregate VOCs only) |
| Strengths | Fast, selective for known targets (PTR-MS), handheld alarms (IMS) | Broad-band spectra, field-rugged, hundreds of wavelengths in one scan | Compact and sensitive for select gases | High resolution/speciation, trace-level detection | Simple operation, low cost | High sensitivity to total VOCs |
| Weaknesses | Limited multiplexing, bulky pumps (except IMS), cross-ion interference | Complex optics (DOAS), per-species lasers (TDLAS/CRDS), interference issues | Limited multiplexing, overlap deconvolution | Not continuous, bulky, slow temporal response | Single-compound only, no speciation | No compound specificity, only total VOC signal |

**Table S3.** Abbreviations and Full Names of Gas-Stream Analysis Techniques

| Abbreviation | Full Name |
| --- | --- |
| PTR-MS | Proton Transfer Reaction–Mass Spectrometry |
| SIFT-MS | Selected Ion Flow Tube–Mass Spectrometry |
| CIMS | Chemical Ionization Mass Spectrometry |
| IMS | Ion Mobility Spectrometry |
| FTIR | Fourier Transform Infrared Spectroscopy |
| TDLAS | Tunable Diode Laser Absorption Spectroscopy |
| CRDS | Cavity Ring-Down Spectroscopy |
| DOAS | Differential Optical Absorption Spectroscopy |
| PAS | Photoacoustic Spectroscopy |
| GC | Gas Chromatography |
| NDIR | Non-Dispersive Infrared |
| PID | Photoionization Detector |

Fig. S1. Stainless-steel tube fittings and unions diagram.

**A**

**B**

Fig. S2. Fitting without insulation (A), fittings with insulation (B).

Fig. S3. Example of the field setup collecting data from a single fermenter in a working ethanol plant.
